# Orthopedist involvement in the management of clinical activities: a case study

**DOI:** 10.1186/s12913-021-06299-2

**Published:** 2021-04-01

**Authors:** André Côté, Kassim Said Abasse, Maude Laberge, Marie-Hélène Gilbert, Mylaine Breton, Célia Lemaire

**Affiliations:** 1 Centre de recherche en gestion des services de santé, Université Laval, Québec City, Canada; 2 Faculté des sciences de l’administration (FSA), Université Laval, Québec, QC G1V 0A6 Canada; 3 Centre de recherche en soins et services de première ligne- Université Laval, Québec, Canada; 4 Centre de recherche du centre hospitalier de l’Université Laval, Québec, Canada; 5 Centre de recherche intégrée pour un système apprenant en santé et services sociaux, Québec, Canada; 6 Département d’opérations et systèmes de décision|, Faculté des sciences de l’administration (FSA) Université Laval, Québec, QC G1V 0A6 Canada; 7 Université de Sherbrooke, Longueuil Campus, Sherbrooke, Canada; 8 EM Strasbourg Business School, Université de Strasbourg, HuManiS (UR 7308) Strasbourg, France

**Keywords:** Care management, Physician incentive, Patient outcomes, Quality of healthcare, Orthopedic surgeries

## Abstract

**Background:**

The rapid shift in hospital governance in the past few years suggests greater orthopedist involvement in management roles, would have wide-reaching benefits for the efficiency and effectiveness of healthcare delivery. This paper analyzes the dynamics of orthopedist involvement in the management of clinical activities for three orthopedic care pathways, by examining orthopedists’ level of involvement, describing the implications of such involvement, and indicating the main responses of other healthcare workers to such orthopedist involvement.

**Methods:**

We selected four contrasting cases according to their level of governance in a Canadian university hospital center. We documented the institutional dynamics of orthopedist involvement in the management of clinical activities using semi-structured interviews until data saturation was reached at the 37th interview.

**Results:**

Our findings show four levels (Inactive, Reactive, Contributory and Active) of orthopedist involvement in clinical activities. With the underlying nature of orthopedic surgeries, there are: (i) some activities for which decisions cannot be programmed in advance, and (ii) others for which decisions can be programmed. The management of unforeseen events requires a higher level of orthopedist involvement than the management of events that can be programmed.

**Conclusions:**

Beyond simply identifying the underlying dynamics of orthopedists’ involvement in clinical activities, this study analyzed how such involvement impacts management activities and the quality-of-care results for patients.

**Supplementary Information:**

The online version contains supplementary material available at 10.1186/s12913-021-06299-2.

## Background

Physicians are encouraged to take on a much more active role in the management of clinical activities in hospitals, given their role in the workflow [1–3]. Over 80% of hospital operations are clinical acts prescribed by an attending physician [4]. Physicians have a significant impact on the allocation of resources and, consequently, on the performance outcomes of health care institutions. They are therefore increasingly called upon to become involved in the management of clinical activities [1, 5]. Non-physician hospital administrators, who do not have the necessary authority to impose and control physicians’ clinical activities, have to seek these professionals’ cooperation in order to coordinate all the human, material, and financial activities under their responsibility.

Physicians’ involvement in the management of clinical activities in health care institutions is therefore of high importance, as the cost, quality, and relevance of patient care depend on the attitude, actions, and level of their cooperation with other health professionals [6]. Research shows that physician involvement in the management of hospital clinical activities is critical to the safety and quality of care [7, 8]. The term “involvement” is used here in its generic sense: to become or be involved as part of the design and operationalization of an action or set of actions. This is similar to the notion of “social interaction*”*, as defined by Goffman [9], “the reciprocal influence of individuals upon another’s actions when in one another’s immediate physical presence” (p.14)*.* For Turner, interactions are a way of showcasing actors’ resources so that the exchange will result in a mutually beneficial situation [10].

Specifically, physicians’ involvement in the management of clinical activities refers to the programming (e.g., design and operationalization) and coordination of the care provided to patients. Such active involvement and interactions help develop a collaborative culture in patient care management. Physician involvement in the management of clinical activities in hospitals has been associated with increased efficiency [11, 12], improvement in work practices and underlying innovations [3, 13] and in the quality and safety of the clinical acts performed [1].

However, despite such benefits and efforts made, institutional, structural, and situational factors seem to make physicians wary of getting involved in the management of clinical activities in their hospitals. Resistance and reluctance to get involved might be due to differences in their social identities that consider physicians as autonomous individuals within the public health system and managers as subordinates to the organizational system [14]. Psychological analyses suggest that the physician identity is stronger among physicians who are managers than the non-medical manager identity; this would explain why physicians tend to generally remain first and foremost physicians [14]. The identification with their profession is such that Collins-Nakai [15] goes so far as to call physicians who take up managerial roles “accidental leaders” (p. 68). However, the literature suggests that some physicians consider their involvement in management as rewarding, exciting, and even essential to improving health care processes [15, 16]. Yet, a number of constraints often pose a challenge. From the outset, physicians consider patients to be under their responsibility and not under that of the health care institution. Consequently, they place their relationship with the patient at the heart of care delivery and consider it legitimate to assume effective control over the surgery that arises from this relationship [2, 3, 16]. As clinically and legally responsible for patients, physicians tend to dictate guidelines to other clinical staff members who then carry them out [17, 18]. They are therefore reluctant to participate in care management approaches or other interdisciplinary teamwork that requires sharing clinical decision-making [19].

Other challenges are structural: formalizing roles, individual physicians’ personality traits, and clarifying mandates. However, any collective action requires negotiation (or renegotiation) of roles, rules, status, expectations, and privileges [20]. Physicians are interested in taking on the management role if it is likely to improve their clinical interventions and reduce the number and length of patient procedures [1]. Therefore, focusing on a common goal, such as the quality of care, might reduce conflict between clinical and managerial objectives and interests. Tension is often accentuated in a context where other professionals’ workloads are heavy and a shortage of medical staff is recurrent [1]. Time is a limited resource and therefore is an obstacle to physicians’ participation in managing their institution [19, 21]. In addition, managerial tasks already occupy a considerable amount of clinical time, resulting in increased patient wait times for consultation or surgery and physicians feel a stronger obligation to patient care [16].

Lastly, lower remuneration might be a structural hindrance to their participation. In Canada, for instance, since physicians are generally paid on a fee-for-service basis, their incomes would be affected if they performed fewer procedures in order to contribute to management activities [1]. Physicians who take on managerial role may be perceived negatively by their peers [7]. They are often considered by their peers to be “double agents” claiming loyalty to their medical vocation [2, 3] but also protecting resources via their managerial role [22].

The other impediments to physicians’ involvement in management activities are organizational and institutional. Physician-managers deplore a lack of general support from their health institutions [1, 13]. The constant quest for efficiency would seem to stir disillusionment among physician-managers in respect to the possibility of participating in truly improved care delivery, given that the demands are particularly high and the resources available are often too limited [8]. In addition, bureaucratic and organizational complexity often does not allow for any real work efficiency, and is sometimes even counterproductive, if inappropriate and uncoordinated initiatives are implemented [3, 16]. Physicians also perceive an overall absence of recognition, which manifests as clinical team leaders’ lack of positive and useful feedback, encouragement, and consideration of their particular needs and interests [2, 3, 5, 8, 16].

Healthcare institutions seem to often find themselves in a paradoxical position. Efforts are made to involve physicians in the management of clinical activities, yet the very nature of the medical profession, the organization of medical practice, and other organizational and bureaucratic obstacles dissuade physicians from doing so. Mintzberg [23] considers that, to build genuine systems that both promote health and treat illness, we need cooperation, not competition (p.44). For this author, ultimately, care, cure, control, and community have to work together within and across health-care institutions to simultaneously deliver quantity, quality, and equality. One study suggests that more empirical research is needed to explain the processes and practices used by organizations and systems to engage physicians [1] in managerial roles.

Understanding the underlying factors of the dynamics is key to improving health care systems as a whole. This study contributes directly to furthering the understanding of these dynamics and proposes solutions to bridge the gaps for a better involvement of physicians in the management activities of orthopedic care pathways. This study aims to analyze and explain the underlying dynamics of physicians’ involvement in the care pathways of orthopedic surgeries. More specifically, it aims to: (1) highlight the different levels of orthopedists’ involvement in the daily management of clinical activities; (2) analyze the impact of such involvement on the flow of clinical activities; and (3) identify the main response deployed by other health care workers related to orthopedists’ involvement or non-involvement in clinical activities.

## Methods

We used a multiple embedded case studies approach [24] to examine how orthopedic surgeons engage in managerial roles.

### Study setting

For the purposes of this study, we considered the care pathways of the three orthopedic surgeries—hip fractures (HF), total knee replacements (TKR), and total hip replacements (THR), at the Centre Hospitalier Universitaire (University Hospital Center (hereafter, “the UHC”). Together, the three selected care pathways account for more than 70% of orthopedic activities and surgeries performed in four out of the five teaching hospitals that compose the UHC, which is located in Quebec, Canada. The UHC has 13,115 employees (1300 physicians and 512 scholars) and an annual budget of 1.2 million US dollars. Each site has its own managerial structure. At three sites (A, B, and C), orthopedists work independently of one another, whereas at the other two (D and E), most physicians work in interdisciplinary teams alongside other types of health care providers, as presented in Table 1. These realities arose from mandatory mergers of A, B, and C, and of D and E in the mid-1990s. All five sites merged voluntarily in 2012 to become a single administrative structure.

**Table 1:** Distribution of Surgeries at the Four Sites and List of Interviewees

	Number of surgeries performed between April 2013 and March 2014^a^			
	Site A	Site B	Site C	Site D
**Pathway**				
Total knee replacement (TKR)	133	142	52	467
Total hip replacement (THR)	137	105	–	313
Hip fracture (HF)	364	–	–	301
**Interviewees**				
Operating block (OB) coordinators	1	1	1	1
Operating block assistant head nurses	1	1	1	1
Nurses (surgical first assistants or clinical nurses)	2	1	1	1
Assistant (OB) coordinators for preoperative care and/or ambulatory care units	1	1	1	1
Post-operative clinical unit heads	1	1	1	1
Assistant head nurses (AHN) for postoperative care	1	2		1
Physiotherapists	1	1	1	1
Social workers and/or the liaison nurse	1			1
Orthopedists	2	1	1	2

^a^*Arthroplasties primaires et fractures de hanche 2013–2014, Direction des finances - Gestion de l’information, performance et mandats spéciaux*, 2015-01-31. Source: MED-ÉCHO

### Data collection

Data were collected between 2014 and 2016. Two data collection techniques were used: a review of the organization’s documents and semi-structured interviews. About 500 pages of internal documents on the hospitals’ governance and the organizational context of orthopedic surgeries at the four selected sites were examined. These included meeting reports, study reports, care protocols, internal notes, and presentations. Then, semi-structured individual interviews were conducted until data saturation was reached at the 37th interview, so as to include a representative from each group of healthcare providers involved in managing orthopedic care pathways at each of the four sites (Table 1). We structured the interview guide based on Kruijthof’s questionnaire [25], adapting it to fit our research purposes and the context of orthopedic surgery activities. We also modified the questionnaire so that it could be used to interview any clinical staff member involved in the orthopedic care pathway. As presented in the Table 2 and Supplementary Interview Guide S1, the semi-structured interview was designed to cover three main parts: (1) the governance (the administrative organizational structure, the mechanism of coordination, the orthopedists’ levels of communication and interaction, etc.) at each of the four sites; (2) the orthopedists’ roles and perceptions of their work; and (3) the orthopedists’ involvement in the management of clinical activities. These activities were categorized into five steps of the orthopedic care pathway: (i) the daily surgery schedule; (ii) instrument management; (iii) patient preparation; (iv) the preoperative phase; and (v) the postoperative phase. Indeed, these episodes were created after fully reviewing the four sites’ internal documents and the literature. Clinical activities associated with Episode 1–4 took place in operating rooms (OR), while postoperative activities (Episode 5) occurred in the orthopedic care unit.

**Table 2:** Levels of Orthopedists’ Involvement in Clinical Activities, by Episode of Care

Clinical Activities, by Episode		Site A	Site B	Site C	Site D
**Episode 1: Daily Surgery Scheduling**					
Submission of surgery requests		Reactive	Reactive	Reactive	Reactive
Follow-up on surgery requests		Inactive	Active	Inactive (parallel management for the permanent orthopedist)	Contributory
Participation in surgery scheduling					
Management of problem cases					
**Episode 2: Instrument Management**					
Equipment orders and delivery		Inactive	Active	Active	Contributory
Transmission of information					
**Episode 3: Patient Preparation**					
Hip fractures	Medical consultation requests	Active or Inactive	n/a	n/a	Contributory
Elective cases (TKR/ THR)	Patient evaluation	Inactive	Contributory	Inactive, except contributory for the full-time orthopedist	Contributory
	Complex case management				
	Transmission of information				
**Episode 4: Intraoperative Phase**					
Surgery time management		Reactive or Inactive	Contributory	Reactive	Contributory
Work organization in the operating block					
Clinical practices improvement					
Daily management of clinical activities		Reactive	Contributory	Reactive	Contributory
**Episode 5: Postoperative Patient Follow-up**					
Routine medical visit		Inactive	Contributory/Active	Inactive, except active for the full-time orthopedist	Contributory/Active
Patient discharge					
Intervention in the event of complications					
• **Inactive:** Not involved in developing management methods and of response to directives and requests • **Reactive:** Available if needed and responds to requests • **Contributory:** Contributes to activities to develop management and intervention methods, and delegates application to other clinical staff members; however, orthopedist keep a close eye on planned activities and intervene if necessary • **Active:** Shows active participation in the development and application of management methods					

### Data analysis

Our analysis of the data was based on the progressive method developed by Miles and Huberman that combines the advantages of a structured approach with the rich and exploratory perspective of a case study [26]. This allowed hypotheses to emerge from the collected data. Orthopedist involvement and impact in the orthopedic care pathway was evaluated at the five clinical episodes, as perceived by the clinical staff. Measurement was focused on orthopedists’ self-reported perception of the impact of their involvement and on other healthcare workers’ responses. Interviews were recorded and lasted for about 55 min each. They were transcribed into a Microsoft Word document and analyzed using QSR-NVivo 11 software. The data from the interviews were rounded out with the literature to map out orthopedist involvement in the management of clinical activities at the four hospital sites.

### Ethical considerations

This study was approved by the Institutional Review Boards of University Laval and Medical Research Ethics Committee of Centre Hospitalier Universitaire (CHU) Quebec Canada, with the ethical approval registration number (2015–2182). Informed written consent was obtained from all participants prior to the start of the interview. Information obtained at the individual level was kept strictly confidential.

## Results

We analyzed orthopedists’ involvement in management, explained variations according to the activities carried out during the five episodes of the care pathway, and determined the impact of such involvement on the flow of clinical activities. We identified four levels of involvement, as shown in Table 2 (more details in Table S2) classified from the lowest to the highest. The lowest level of involvement was inactive involvement, which is characterized by the lack of orthopedist involvement in clinical activities and response to directives and requests. However, the level became reactive involvement when the orthopedist was available, if needed, and responded to requests. When orthopedists contributed to management activities and delegated some activities to other health care providers, keeping a close eye on planned activities, and intervening when necessary, their involvement became contributory involvement. Lastly, active involvement was characterized by orthopedists’ active participation in the development and application of clinical management activities.

The underlying dynamics of orthopedists’ involvement were examined and compared during the five episodes of the care pathway at all four hospital sites. The implications of such involvement were also evaluated and compared to indicate the main responses of other health care providers to each episode at the four sites, as described in detail below and summarized in Table 2 (more details in Table S2).

### Descriptive and comparative analysis of the organizational context and the impact of orthopedist involvement at the four sites

#### Episode 1: daily surgery schedule

Orthopedists’ involvement was different at each of the sites in this case study, depending on the hospital’s organization of its medical practice. At Site A, most orthopedists paid little attention to procedures and had limited knowledge of patients’ pre-surgery preparation. Since they had only a vague grasp of their surgery schedule, they regularly added elective cases (TKR & THR) at the last-minute without being aware of the consequences of these additions. This situation could be explained, in part, by the control that orthopedists had over their surgical practices, and also by institutional rules that allowed them to determine and prioritize up to 50% of the cases to be included in each orthopedist’s daily schedule. Some even considered that they should be entitled to more control: “I should be 100% responsible for the operating day. Management doesn’t know my patients. The managers want more and more control over what happens between my patients and me. I can’t accept that*”* (A9). Similarly, the orthopedist at Site C drew up the surgery schedule according to their personal availability and sent it to the assistant head nurse (AHN) in charge of the surgery schedule.

Meanwhile, orthopedists at the other sites (B and D) were more involved in management activities. At Site B for instance, orthopedists were available if needed for most of the daily clinical activities, thus facilitating communication with other members of the team. However, the orthopedists at Site D had contributed to developing institutional regulations governing access to surgery. These stated that the surgery schedule was managed by a centralized surgery planning unit. To prioritize their surgery requests, the orthopedists at this site used a priority scale of P1 (most urgent) to P4 (least urgent). The centralized surgery planning unit scheduled the surgeries based on the priority level of patients. On their surgery requests, the orthopedists also indicated the medical consultations required during patient preparation. They were accessible, had a fairly accurate idea of the cases waiting for surgery, and maintained smooth communication with other members of the health care team.

The lack of involvement of the orthopedists at Sites A and C had a number of consequences, including a high number of last-minute requests, which caused many delays and postponements for elective joint surgeries (TKR and THR). This, in turn, negatively affected the work of other clinical staff members. For instance, the assistant head nurse (AHN) had to change the order of priority, call other patients, or even cancel surgeries. As one nurse expressed, “Our workload is doubled or tripled” (A2). Consequently, some patients have to come back to the pre-operative clinic and redo required tests and consultations, if the timeframe has been exceeded. Inversely, orthopedists’ involvement in planning activities at Sites B and D facilitated operations and had a number of advantages. For instance, at Site B, constant communication between the AHN responsible for the surgery schedule and orthopedists might prevent the negative consequences observed elsewhere.

When an unforeseen situation arose, the AHN changed the schedule, on the assumption that the orthopedists would approve the adaptation. At Site D, last-minute requests were reduced by designating a specific time slot for elective joint surgeries and another for emergency cases (namely, hip fractures). Unless there was a life-threatening emergency, last-minute cases did not disrupt the daily schedule. Furthermore, the centralized surgery planning unit at Site D facilitated the selection of patients who were already prepared for surgery, in the case of interchanged patients or cancellations.

#### Episode 2: instrument management

Orthopedists at Site A only glanced at instrument orders and did not follow up on them. At Sites B and C, however, on-site orthopedists submitted the order for their prostheses themselves and then double-checked upon reception that the orders had been correctly filled. At Site D, instrument management was incorporated into the centralized surgery planning protocol. Given their sense of ownership as co-developers of the protocol, orthopedists were vigilant in ensuring that all procedures were compliant, which in turn was greatly facilitated by having a centralized protocol.

It was observed that the impact of orthopedists’ lack of involvement on the flow of operations was mitigated by other clinical staff members’ interventions. At Site A, for instance, the AHN responsible for material resources found work-arounds for instrument management. To prevent errors in the ordering and preparation of instruments, which could result in the cancellation of procedures, the first surgery assistant documented each orthopedic surgeon’s operating procedures. Working with each instrument supplier, the RN-surgery created a form with insets containing specifications for the various prostheses in use. The RN-surgery’s knowledge of operating procedures also supported the work of orthopedists, nurses and the AHN for material resources. Moreover, the RN-surgery identified problems and suggested appropriate corrective actions. The RN-surgery could also spearhead efforts to review orthopedists’ operating procedures and oversee OR nurse training.

At Site B*,* a series of charts were drawn to depict the types of prostheses used by each orthopedic surgeon. Completed forms were also sent to the sterilization and purchasing departments indicating each orthopedic surgeon’s requirements for each type of procedure. Similar initiatives were also taken at Sites C and D.

#### Episode 3: patient preparation

Two patient preparation situations were studied: hip-fracture (HF) cases treated at Sites A and D, and elective-joint surgeries (TKR and THR) conducted at all four hospitals. At Sites A and D, HF patients were normally managed by an emergency orthopedist who referred them to the orthopedic surgeon on duty. However, at Site A, while some orthopedists validated that a consultation had indeed taken place, others simply submitted a surgery request on the assumption that the routine consultation had already occurred. This was complicated when patients were referred from other emergency rooms, since they may not yet have been prepared for surgery.

At Site D, hip fractures were managed ahead of time by emergency orthopedists on the trauma team. The orthopedists at Site A worked independently, unlike those at Site D who managed patients as a medical team. In fact, the orthopedists at Site D came together under a medical group management structure (pool) that handled all health care workers’ income, redistributed sums as agreed. Proposed innovations were carefully examined, analyzed, accepted, or rejected as a group.

In the preparation of elective cases (TKR and THR), patients’ medical and pharmacological assessments were carried out by an internist at Sites B and C or by an anesthesiologist at Site D. Under agreements between medical specialists (internists, anesthesiologists, and cardiologists) and orthopedists, specific slots of time were assigned to orthopedic patients at Sites B and D.

Meanwhile, the situation was more complicated at Sites A and C for patients under the responsibility of orthopedists on the mobile team. Since there was no agreement among orthopedists, management fell to the pharmacist on duty, who was called to prepare a medication profile, and to a general practitioner, who was expected to assess the patient and prescribe any drugs. Medical specialists did not assign slots of time for meetings with patients at Sites A and C, and some orthopedists were difficult to reach since they did not have offices on site.

#### Episode 4: the intraoperative phase

During the intraoperative phase, orthopedists’ involvement in the daily management of clinical activities was at the ‘contributory involvement’ level at Sites B and D. At Site D, the medical group management structure, combined with interpersonal dynamics conducive to communication between orthopedists and other clinical staff members, facilitated their involvement. The situation was similar at Site B, where orthopedists were not part of a medical group management structure, but had developed a shared practice of exchanging cases, thus allowing each surgeon to specialize in a particular type of procedure. At these two sites, orthopedists also contributed to the organization and management of work practices by participating in clinical quality-improvement committees.

The orthopedists at Sites A and C were not involved in the committees that managed daily clinical activities. They simply made note of the instructions and orders issued by these committees. As a consequence, they did not feel involved in clinical improvement initiatives. For instance, at Site A, the institution’s senior manager introduced a process for reviewing operating procedures (the Lean Healthcare approach) [5], which received a lukewarm response from orthopedists. They saw the approach as a strategy to force them to adopt methods originating elsewhere.

In sum, we observed that orthopedists’ inactivity in elective surgeries at both Episodes 3 and 4 impacted work organization and patient care. For instance, at Site A, the clinical staff was constantly trying to track down attending orthopedists to obtain necessary medical prescriptions: “It takes an incredible amount of energy to meet with them because they do not have offices” (A1).

Furthermore, last-minute changes to surgery schedules lengthened patient preparation. To avoid pointless consultations, a multi-site interdisciplinary team developed a data collection form to be completed by patients. Data collection was crucial to issuing the required prescriptions for patient preparation and to identify patients whose hospital discharge was anticipated to be difficult. At Site C, the nursing staff had to check that no data was missing for patients transferred from Site A due to bed shortages, and they often had to request that consultations in internal medicine be repeated.

In contrast, at Sites B and C, where orthopedists had a contributory level of involvement, this procedure went smoothly, thanks to the orthopedists’ agreements with internists about preoperative patient assessment and with cardiologists and anesthesiologists about designated time slots for consultations. Intraoperative activities were also incorporated into the centralized surgery planning protocol at Site D. At Site C, the orthopedist also contributed to patient preparation, for instance, by submitting their surgery schedule months in advance, thus allowing nursing staff ample time to prepare patients. As a result, only one case had been postponed in the last 10 years due to missing tests.

#### Episode 5: postoperative patient follow-up

Orthopedists’ involvement in postoperative patient follow-up was subdivided into routine medical visits, interventions in the event of complications, and patient discharge. At Site A, three out of four hip-fracture patients were managed by internists, and patients undergoing arthroplasty may receive a medical visit, depending on the orthopedic surgeon. At Sites B and D, orthopedists delegated this task to orthopedic residents. At Site C, the on-site orthopedic surgeon followed up with arthroplasty patients himself, and as one patient said, “He knows what is happening. He visits his patients and he talks to us…” (C1). At Site D, most hip fractures are referred to general practitioners in the trauma program. Therefore, although they belong to the same professional order and practice in similar settings, orthopedists’ level of involvement in clinical activities varied.

The involvement of most orthopedists working at Site A was either inactive or reactive, while at Site C, it was a mix of inactive and reactive; orthopedist involvement was often active or contributory at Sites B and D. These levels of involvement had an impact on the flow of activities, and on other health care providers, who developed mitigation strategies to ensure that the work proceeded efficiently and to attenuate the effect of low orthopedist involvement on patient care.

At all sites, the in-unit absence of orthopedists for post-operative follow-up required nursing staff to justify consultation requests for each patient. It was difficult for orthopedic residents to compensate for orthopedist absence, since they were neither familiar with patients’ care pathways nor well informed of the procedures in the operating room (i.e., for the surgeries). Hard-to-reach residents were also not available to answer the clinical staff members’ questions and merely entered instructions directly in patients’ medical records. The approach of working in silos also affected the discharge process. Orthopedists could write “discharge by physiotherapist” (D3).

However, physiotherapists were not all comfortable taking this responsibility, particularly when there was a complication. They would then delegate the discharge authorization to internists (Sites A and C) or to residents (Sites B and D), which caused further delays. For hip fractures, the discharge decision was often made through interdisciplinary collaboration with the geriatrician or internist at Site A, while at Site D, general practitioners in the trauma program and residents authorized discharges. To offset the absence of orthopedists, clinical staff members attempted to formalize and standardize care processes by developing a number of clinical tools, including care pathways profiles and preprinted individual prescriptions, the success of which varied by site. At Site A, care pathways were reviewed in the early 2010s, although this does not appear to have been sufficient: “The entire chain would have to be reviewed. But that would also mean surgeons would have to be willing to be in charge of it” (A3).

At Sites B and C, care pathways no longer reflected current averages for the length of hospital stays. Given they had not been updated and new staff members were not encouraged to use them, these care pathways profiles were not very useful. At Site D, an interdisciplinary team updated the care pathway. A proactive patient discharge criteria form was created to promote follow-up on episodes of care. However, this form became a checklist entered as standard practice or out of habit into patient records; as a result, a number of activities were neglected, including the patient education component. This was also the case with the short care pathway at Site B.

A nursing care and treatment plan (NCTP) that included a care pathway was also created at Sites A and B. However, this tool was not always updated daily by staff members as it should have been to reflect personalized patient care. At Site D, care providers used a traditional NCTP for hip fractures that did not include a care pathway and electronic records that contained various care providers’ notes. Orthopedists from Site D regularly used preprinted individual medical prescriptions. Lastly, health care providers also emphasized the importance of a standardized approach. By constantly repeating “the patient has to leave in four days” (D3), they were aware of the importance of reviewing practices to achieve this discharge objective. The secret was to involve health care providers and provide them with constant reinforcement. Lastly, a patient coordinator was also integrated into the patient care unit at Site A.

## Discussion

This study showed that orthopedists do not have to be equally involved in each aspect of clinical activity management. Engaging in management activities is an important component of these orthopedists’ work to improve the flow of care pathways. This study highlights the varying levels of involvement among orthopedists in management activities. TKR and THR are considered as having very uniform care pathways profiles [27–29]. Consequently, these procedures do not require orthopedists to be involved at every stage of the care pathway. The routine nature of certain tasks means that a number of decisions can be programmed as part of formalized and standardized care protocols. Hence, our study showed that orthopedists’ involvement is desirable and necessary in the development and updating of protocols and preprinted individual prescriptions, but not necessarily in their daily application.

Under particular circumstances, such as complex cases, emergencies, or the management of unforeseen events, orthopedists should play an active role in both decision-making in clinical activities and in implementing such decisions; or, at least, they should agree with other specialists to ensure proper care management of these cases. Regardless, their involvement is essential to ensuring smooth patient care. Empirical evidence [7, 13, 16, 30] indicates how the involvement of orthopedists can be encouraged in the management of clinical activities. This study identified valuable strategies for fostering orthopedist involvement, presented in the following section.

### Activities for which decisions cannot be programmed in advance

As noted earlier, because orthopedists consider patients as being under their responsibility and not that of the healthcare organization, it can be challenging to have them collaborate with other healthcare staff members. Thus, control over and autonomy in their medical practices are fundamental elements of orthopedists’ interventions. Formalizing exchanges with team members are important to develop a collaborative work culture. Turner (2003) sees an interaction as a way of showcasing an actor’s resources so that exchange will result in a situation that is of benefit to this actor. In this case, we found orthopedists are willing to cooperate in any initiative that will make their clinical procedures more effective. Indeed, the notion of continually improving the quality of clinical procedures is very important in the medical culture [16]. The opportunity to meet the specific expectations of the actors concerned is also for orthopedists to engage in exchanges. The possibility of reducing orthopedists’ workload, speeding up the procedure and care episode, and increasing the number of paid medical procedures are also reasons that could foster orthopedists’ collaboration.

Interacting with other members of the health care staff, like repeated discussions among partners in the same space and time, has a reciprocal influence on stakeholders’ respective actions [10]. Regular daily or weekly meetings to coordinate staff activities in certain operating rooms (including those of orthopedists) were excellent opportunities for interaction. The same was true in orthopedics departments, where multidisciplinary meetings attended by all healthcare providers led to more effective decision-making [31].

The presence of a specialized resource also created a number of opportunities for regular interaction with orthopedists. The example of the first RN-surgery assistant at Site A showed that specialized resources can introduce new tools and methods of intervention, periodically update clinical practices, and ensure these are applied on a daily basis. Since orthopedists are often reluctant to attend clinical training and have considerable difficulty working as part of an interdisciplinary team [12], the organization of workshops will be conducive to dialog between the team of healthcare providers and orthopedists that the latter will take an interest in nurses’ recommendations.

Our study also showed that the standardization of care facilitates interactions with orthopedists. Strauss et al. indicate that any collective action requires a negotiation or renegotiation of roles, rules, status, expectations, and privileges [20]. This negotiation can be very difficult when it involves a wide range of actors working individually, who are motivated by specific expectations and interests, and who are not governed by any formal relationship of authority. The medical group management structure at Site D simplified this negotiation process by regulating expectations and interests for their members and by gradually introducing rules and agreements that govern both the department’s operations and the organization of medical work as a whole.

Furthermore, it would seem that this group structure encouraged orthopedists to standardize their clinical practices and discuss ways to improve them. The homogeneity of the medical practices made introducing innovation easier. Clinical teams also compelled orthopedists to reflect collectively and thus led to the adoption of a common position. Orthopedists’ involvement in the organization’s management processes was thus facilitated, since the group delegates a spokesperson to participate in the development of management methods and ensures that these are applied by all.

The study shows that the level of involvement of orthopedics influences the work of others professionals thus playing a role in the implementation of innovations. The contributor involvement helps notably the inclusion in the groups in the patient preparation episode. The active involvement facilitates the implementation of the innovation, through further training and reading as identified in the daily surgery scheduling episode (Table S2). An indirect influence is also identified for inactive and reactive involvements that prevent professionals from implementing innovations. In the management episode instrument, the inactive and reactive involvement is compensated by middle managers (Site A), while they could have developed innovations. In line with Birken’s theory of the middle managers’ role in healthcare innovation implementation [32–34]. The results show the importance of implication of all healthcare professionals and their coordination to implement innovations [35]. They also confirm that trust in management has a cascading influence across the levels in the organization [36].

The various initiatives by senior management and the professional services department to involve orthopedists in transformation processes via routine discussion of their respective practices gave rise to a formalization and standardization of medical practices. In formal meetings, orthopedists were called upon to adopt the most effective practices. By participating in the collective decision-making process, they implicitly agreed to comply with group decisions.

Cooperation also took the form of formal agreements among various specialized health care providers, including orthopedists, which facilitated the work of the entire healthcare team. For instance, formal agreements between orthopedists and other medical specialists regarding the management of certain steps in the care pathway showed that the work can be distributed in such a way that each health care provider performs the appropriate procedures in a timely manner, thus preventing duplication or delays in the continuum of care. However, formal exchanges alone were not sufficient to get orthopedists involved in the management of clinical activities. Grémy [37] found that medical partnerships do not work unless formal discussions are combined with informal exchanges between the orthopedists and other clinical staff members.

This study also showed that orthopedists’ regular presence at the hospital site allowed for ongoing communication with other healthcare providers. In patient care units where the desired outcome required frequent adjustments between care providers, the quality and assiduity of exchanges is equally—if not more—important than formalized methods of intervention. For instance, even though there was no well-defined procedure for orthopedic patient preparation and follow-up at Site C, the care pathway was nonetheless very smooth for patients under the responsibility of the orthopedic surgeon who practices there full-time. However, infrequent exchanges meant that clinical activities did not flow nearly as smoothly at Site A where other care providers’ concerns and expectations were considered and integrated only sporadically.

### Activities for which decisions can be programmed

Clearly, all decisions are not of the same importance, nor do they all require a high level of orthopedist involvement. However, orthopedists who absolutely wished to maintain their control (i.e. an exclusive right to decide who is authorized to carry out tasks and how [18]) did not find it easy to adhere to programming the clinical activities under their responsibility. In this regard, a number of authors highlight the importance of trust [6, 13, 22] in a relationship based on shared values, the definition of collective concerns, and the standardization of productive practices. Consequently, trust is a tool for predicting action and achieving objectives. For instance, orthopedists at Site D allowed nurses to prescribe certain medications, which reduced their control but facilitated day-to-day work by making the nursing staff more autonomous.

However, one of the key elements in building trust-based relationships is the stability of clinical staff. Indeed, it is difficult to propose and implement a new idea if there is high turnover within the team. Turner [10] also impedes the sustainability of clinical practice management structures. When not encouraged to apply and update patient care pathway protocols, new staff members tend to fall back on old ways. Frequent changes in the healthcare staff force orthopedists to invest additional time and energy in building and rebuilding communication. This situation was all the more challenging when patient navigators who regularly interact with medical professionals leave. Some researchers have examined ways to stabilize work teams [38–40]. The ‘magnet hospital’ is one interesting and inspiring model. The factors of its success are attracting and retaining nursing staff through the interactional dynamics of healthcare teams and professionals’ desire to grow and develop in such an environment [41, 42].

### Limitations and strengths

This study is not without limitations. First the semi-structured interviews for data collection could not exclude the likelihood of bias, which could have influenced the findings. Second, this study was limited to a single organization and one medical specialty, and thus our findings are most likely a representation of the situation of that organization. Despite the above limitations, the primary strength of this study is the advantageous applications of rigorous semi-structured interview and content analysis techniques to assess our specific study aims. To the best of our knowledge, this is the first study using semi-structured interviews approach to analyze and explain the underlying dynamics of physicians’ involvement in the care pathways of orthopedic surgeries, as well as investigate the quality-of-care results for patients. Unlike large population surveys, semi-structured interviews allowed in-depth and nuanced explorations that gained a rich narrative of data from a relatively modest sample of regarding their perceptions of orthopedists’ involvement in clinical management activities. Furthermore, we can conclude that the level of orthopedists’ involvement in clinical activities has an impact on the healthcare organization, as well as on patient health outcomes. This could serve as useful inputs for developing management practices that take medical formalized protocols into account.

## Conclusions

Our results show orthopedic surgeons have various levels of involvement, ranging according to care episode from the lowest to the most active of levels. Our research also illustrates that their involvement in the day-to-day management of care need not be of the same nature, and in fact depends on the patient care activity being conducted. By considering how orthopedic involvement impacts the work of other health care workers and the strategies these latter deploy, among other aspects, our research highlights the difference in involvement between activities requiring medical decision-making that is not easily preprogrammed and decisions can indeed be programmed. Based on our findings, we would recommend that orthopedist involvement in decision-making of clinical management activities be encouraged in complex cases and in emergency cases. For elective cases (TKR & THR), the orthopedists’ involvement is desirable in the development and updating of protocols and preprinted individual prescriptions, but not necessarily in the daily activities. The collaboration and partnership underlying physician involvement depend neither solely on the organizational structures put in place nor solely on the relationships built between actors. Since orthopedics and other health care providers have a wide a variety of interactions about information to be shared, objects or instruments to be used, samples to be taken, or services to be rendered, it would indeed appear that the relationships that must be built and maintained between stakeholders are just as crucial as mechanisms and structural modalities.

## Supplementary Information

**Additional file 1.** Structured Interviewing Guide.

**Additional file 2: Table S2.** Levels of Orthopedic Surgeons’ Involvement in Clinical Activities, by Episode of Care and Other Clinical Staff Members’ Responses.

## Data Availability

Additional datasets used and/or analyzed during the current study are available from the corresponding author on reasonable request.
