# Supplementary material for: Orthopedist involvement in the management of clinical activities: a case study
Source: BMC Health Serv Res. 2021 Apr 1;21:299. doi: 10.1186/s12913-021-06299-2 (PMC8017788; doi:10.1186/s12913-021-06299-2)
Supplement: Supplementary file 1 — Additional file 1. Structured Interviewing Guide. [file 12913_2021_6299_MOESM1_ESM.pdf]

Structured Interviewing Guide

Impact of the Orthopedist Involvement in the Management of Clinical Activities at Centre Hospitalier Universitaire (University Hospital Center, CHU**) in Quebec, Canada**

This academic research project aims to document and analyze the dynamics of orthopedist involvement in the management of clinical activities for three orthopedic care pathways including the hip fracture (HF), total hip replacement (THR) and total knee replacement (TKR) within different hospitals centers in Quebec, Canada. In doing so, the project analyzes the implications of such involvement and indicated the main responses of other health professionals. Four surgeries sites (Centre Hospitalier de l'université Laval (CHUL), Hôtel- Dieu de Québec (HDQ), Saint-François-d'Assise (HSFA) and Enfant-Jésus (HEJ)) have been selected for the purpose of this study and semi-structured individual interviews had been conducted to evaluate the impact of orthopedist involvement in the management of clinical activities. For the purpose of this project, this structured Interviewing Guide was developed to provide an overview of the methodology utilized to guide interviewers and participants, and described the main objectives of this project. Mainly, the semi-structured interview was based in four objectives, as described below:

The observations gathered during the interviews will be analyzed according to four main objectives, as described below:<br>**Objective1:** The functions of the various actors involved in each of the care episodes.<br>**Objective2:** The different tasks and activities carried out in each of the care episodes, the modes of action<br>**Objective3:** The interactional dynamics between the actors operating within each episode of care and between the episodes of care, the interaction routines.<br>**Objective4:** Opinion on the variation of length average of stay within different hospitals and the impact of orthopedist involvement in the clinical activities.

The sections of the interview will be detailed in this guide in order to allow you to seek the maximum amount of information corresponding to the criteria sought for each objective.

Section 1

Observation allowing the analysis of objective 1 according to the following criteria:<br>• The actors involved: The units, departments, specialties and categories of professionals involved.

The participants are pivotal stakeholders in the care process, including administrators, orthopedists, clinicians, physicians, nurses etc. This section will collect personal information as well as the different functions and role of each one participant according to the hospital, the surgery and the episode of care, as presented below:

Personal Information			
Date:	____________	Location:	____________
Employee name:	____________		
Function:	____________	Service:	____________
Supervisor:	____________		

Hospital	Surgery	Care Episode
CHUL ☐	Total knee replacement ☐	Preadmission ☐
Enfant-Jésus ☐	Total hip replacement ☐	Preoperative ☐
St-François d'Assise ☐	Hip Fracture ☐	Perioperative ☐
Hôtel-Dieu de Québec ☐		Postoperative ☐

The desired objective of this section will be meet if the number of participants interviewed were between 5 and 6 in each site. The appendix below presented in details the categories of health personals to contact for the interview and some suggestions of questions.

St-François d'Assise (PTG, PTH, FH)	Enfant-Jésus (PTH, FH)	Hôtel-Dieu de Québec (PTG, PTH)	CHUL (PTG)
**Preadmission**	**Preoperative**	**Perioperative**	**Postoperative**
Coordinating nurse or clinician			
Head nurse	Head nurse	Head nurse	Head nurse
Physiotherapist			
Orthopedists (head of department, head of unit)			
Others as needed			

For the data collection, make a Table for each Hospital and the personal information of participants interviewed.

Examples of questions:

- What is your professional background?
- What is your current specialization?
- What is your professional title?
- Which service are you working?
- Who is your immediate supervisor?
- How is the coordination activity? And management?
- What do you think ?

Section 2

The purpose of this section is to draw a portrait by hospital of the distribution of surgeries.

Please completed the Table below for each hospital

Diversification of clinical activities	Total replacement knee	Total hip replacement	Hip fracture
%			

Section 3

In this section, the purpose the interview will be based to assess the perception and the mode of coordination experienced (positive or negative) by each participant. They will be interviewed for their past and current experiences on the involvement of clinical activities. For instance, make them talk about what they tried to improve the clinical process and what

were the findings. This might lead to understand for example the level of coordination and integration.

Section 4

This section will cover both the objective 2 & 3 concerning the coordination experience and the interaction among health personals. The interview might take about 40 minutes on those issues, as summarized below:

This section will allow to analyze the objective 2 related to the following criteria: the modes of intervention of the actors involved; the technology used, etc. This section will also cover the objective 3, concerning to the following criteria:

- The interaction routines consist of:
- Administrative structures,
- Coordination, cooperation and patient monitoring mechanisms
- Clinical rules or protocols
- Agreements relating to the coordination and execution of tasks or activities, etc.

In summary, it is important to understand well where they are in their coordination and/or they are ready to move towards co-management or not? This guide will make it possible to identify the distinctions between the various coordination, cooperation and patient monitoring mechanisms at various levels. The technologies used, the forms of communication and/or anything that can create good coordination or not among them. And the different levels of coordination activities are presented below:

Speakers	Administrative structures	Coordination mechanism	Rules and protocols	Informal agreement	Interactional dynamics

4.1 Administrative structures

The objective here is to get administrative information (scheduling meeting, planning activities, etc.) for example, every Tuesday morning the orthopedist, anesthesiologist, nurse take the files and peel them and make the calendar of activities: planning of surgeries.

**Examples of questions:**<br>• Do people meet?<br>• Are there formal meeting committees?

4.2 Patient coordination, cooperation and monitoring mechanisms

Sheets, follow-up files are mechanisms. For example, if 12 patients enter an emergency, they send 6 to such a floor and are ready to welcome them.

**Examples of questions:**<br>• Are the processes formalized?<br>• Is it written somewhere how to do it?<br>• Do they know how to do it?<br>• Do others know what others can do?

4.3 Clinical rules and protocols

These are car maps, systematic customer monitoring, intervention protocols. Example of information sought: No matter the doctor, it's the same and everyone knows it. When a patient enters everyone knows it and is ready to welcome him or on the contrary: they tell us nothing, Dr x that's it, and the others do that.

**Examples of questions:**<br>• Do you have formalized intervention protocols?<br>• If yes, is it standardized?

4.4 Agreements relating to the coordination and execution of tasks or activities

An agreement is informal. For example, the communication between such actors is so strong that they don't need to talk to each other. Mutual adjustment, email, other methods. In some hospitals, for example the family doctor is responsible for this. It is important to highlight the modes of coordination presenting facts in order to seek appropriate solutions.

**Examples of questions:**<br>• How people talk to each other to coordinate and schedule their activities.<br>• Who doesn't need to talk to each other to understand each other?<br>• What are the informal modes of coordination?<br>• How do you adjust to each other?<br>• What effective tips have you developed?

4.5 Interaction dynamics

This section corresponds to the level of communication modes. Interaction dynamics are modes of coordination and coordination mechanisms are requests, forms or files. How they speak or do not speak to each other. This part is intended to focus more on the quality of social relationships and personal involvement in the achievement of common objectives. This section also makes it possible to note the peculiarities of social relationships and the stability of clinical teams. This section should allow you to observe the pivotal coordinating actors.

**Examples of questions:**<br>• How do you describe your relationship with the stakeholder involved?<br>• Who is talking to? Who does not speak?<br>• What is the internal dynamic? Ex: staff turnover<br>• What is your motivation to create a change to improve the mode of coordination?
• What mode of coordination (communication method) do you most prefer to use? Is it the most effective?<br>• Is it similar with all stakeholders?<br>• What do you find most difficult in the modes of coordination?<br>• When a particular mode of coordination is imposed following new rules or protocols, what is your reaction?<br>• What are the causes of variations in coordination methods?<br>• When do you sometimes not follow the care plan?

Section 5: Perception of roles and work structures

This section will allow to cover the objective1 according to the following criteria: The actors involved: specialties and categories of professionals involved, units and departments

- Devolved functions, activities and tasks: Example nursing staff: tasks of four orders, namely: (1) the provision of care, clinical practices, (2) management of medical units or records, (3) teaching patients, (4) Role of assistance to medical staff in their clinical research practice or project
- The nature of the tasks performed
- description of the tasks performed
- the skills required for the execution of tasks,
- the degree of identification
- the importance of the task
- the degree of autonomy (independent, interdependent and dependent on the consent of others) etc.
- Their positioning in the care process: Their perceptions, their ideals, their role, their way of proceeding, their degree of autonomy and their contribution within the care process.
- Their understanding of internal logic: Their concerns, their respective expectations, the objectives pursuing, their analyzes of the situation, the lessons they draw from past experiences.

This section will guide also on how to cover the objective2, according to the following criteria:

- the arrangement of specific tasks and activities; care process mapping
- one or more places where tasks and activities take place; space
- the time required for the completion of tasks and activities; management of medical units or records,
- Teaching patients
- Role of assistance to medical staff in their clinical research practice or project
- The nature of the tasks performed:
- description of the tasks performed
- the skills required for the execution of tasks,
- The degree of identification
- the importance of the task
- The degree of autonomy (independent, interdependent and dependent on the consent of others) etc.
- Their positioning in the care process: Their perceptions, their ideals, their role, their way of proceeding, their degree of autonomy and their contribution within the care process.

- Their understanding of internal logic: Their concerns, their respective expectations, the objectives pursuing, their analyzes of the situation, the lessons they draw from past experiences.

This part does not allow the patient's trajectory to be established and a timeline to be drawn in the form of a map. It emphasizes the different interactions and mode of coordination between the stakeholders involved and the time associated with it. Ask for the place to locate in space the moments when coordination is necessary, especially for travel. The role will be approached in a general way: perception, autonomy and the degree of identification will be approached. This part therefore allows us to observe how people perceive the role of other stakeholders in the process, the power they attribute to them as well as their hierarchical relationship with them.

Speakers	Preadmission	Preoperative	Peri operative	Operation	Postoperative

Get people's perceptions of their role in overview. No overflow on the specificity of the tasks, just perceptions on the importance of their role. Talk about their perception, their understanding of their role. How they feel in there and think of others. Understand everyone's tasks and boundaries (maps help when they are done and applied). It is between tasks that is important, coordination. For example, the way nurses undergo modes of coordination. The goal is to bring out the understanding of their task and their role, the way they see themselves in the care process. Position them the way they see themselves: as a support, as a subordinate, a subordinate, a pivotal person. What is their understanding of their role? Understanding this allows us to understand their interaction in terms of coordination modes. For instance, understand the activities to coordinate: my role would be to do that, but in reality I do nothing ... the other does everything and tells us nothing. For example, ‘’ I am a head nurse in principle I should, but in reality I… because… ’’

Examples of questions:

- What level do you intervene in and what do you do?
- What do you do in episode 1, episode 2? At what level of specification? It can vary the time… What is their understanding of your role?
- How do you see your task and the task of others in the modes of coordination?

- What are the factors that influence your efficiency in the care process? (Sense of control, appropriate number of patients, patient characteristics, support from colleagues, appropriate workload)
- For you, what is your most important contribution in terms of coordination mode?
- How do you perceive your decision latitude, your autonomy in decision-making?
- How do you live it?
- Tell me about your perception of your role in the hospital?
- What supports do you use when the task overflows?
- What is your role (if nurse: care, management, teaching, assistance)?
- How do you structure your work?
- What is the role of the stakeholder involved?
- What activities are planned there?
- What activities do you do that are not planned?
- What do you prefer ?
- What guides you in carrying out your tasks? (Personal experience, theoretical model, organizational culture, administrative structures, protocols, informal agreement, unwritten knowledge)
- How independent are you in your tasks? (functions independent, interdependent and dependent on the assent of others)
- How do you perceive the importance of your tasks?
- Who are the stakeholders involved (professional category)?
- What is their specialty?
- How many different people do you do business with?
- Staff turnover, how does this affect your work in terms of coordination methods?
- Preoperative phase
- When the patient arrives in the preoperative phase, are there any variations affecting the smooth running of the planned process? (See if coordination with the pre-admission phase can cause variance) Patient history, request, consultation paper or other.
- Perioperative phase
- When the patient arrives in the perioperative phase, are there any variations affecting the smooth running of the planned process? (See if coordination with the preoperative phase can cause variance)
- Postoperative phase in a hospital environment (stabilization and convalescence)
- When the patient arrives in the postoperative phase, are there any variations affecting the smooth running of the planned process? (See if coordination with the perioperative phase can cause variance)

Section 6: Involvement of physicians in the collective coordination process.

Emphasize the level of implementation of doctors in the collective coordination process. Highlight information such as ‘’ the doctor doesn’t want to get involved’’ to see if there is already a basis for formal authority from doctors over others. For example, with us, nobody makes the decisions, we are not interested, we chat together. This information will give clues on the ease or not of implementing co-management. Make a link with the theory: The position of doctors and multi with regard to organizational and managerial logic. For "Langlay and Denis (2012)" The organizational / managerial logic is based on the principle that it is possible to improve care and services by attaching more importance to the roles that management and organizational practices can play (design by program, multidisciplinary or interprofessional team work, for example), decision-making mechanisms such as setting priorities and objectives, coordination mechanisms, control and accountability mechanisms. " For orthopedic surgeons, have them talk 10 minutes on it if necessary. It’s the heart to know whether they’re ready for co-management or not ready at all.

Examples of questions:

Important to know if doctors are still trapped in this logic or their thinking is changing?

- Are they ready, do they have an opening to this logic? Or if they see it as a terrible danger.
- What is their level of involvement of doctors in decision-making?
- What is their predisposition to get involved? Multi and med
- How do you see co-management?
- To orthopedists: Do you think it would be good to get more involved in a formal mode of a coordination process.
- To the nurses: do you think the doctors and multi are ready?

Section 7: Opinion on the variations of average length stay across hospitals

Document the factors behind the variation of average length stay across hospitals with the orthopedists’ involvement or not in clinical activities. It may be the only question asked to get (by triangulation) information from the grid. To validate previous information. Start with this question for orthopedic surgeons and complete with the other questions if necessary. For example, the number of doctors varies between hospitals which can affect the length of waiting lists. This can be seen in conventions as a family doctor does this and not elsewhere. In addition to the logical explanation, it would be interesting to assess the personal power affects the perception of the person at the level of his decisional latitude,his

autonomy and his own control at the level of the solution (degree of identification and autonomy) . Here, innovative solutions may emerge in terms of modes of coordination.

Examples of questions

How to explain the variations in MDS between hospitals by site and by doctor?

- How to come to the conclusion of why average length of stay is due to the mode of coordination?
- What is the proportion of delays due to the method of coordination versus other variants (patient's condition, technical factor)? General perception.
- Who can handle this kind of situation? At what hierarchical level?
- In an ideal world, what do you think should be done?
- What would be the simplest for you?
- What is your perception of the change in management methods according to your past and current experiences?
- What does it teach you?
- Identify successful situations in terms of coordination methods of which you are particularly proud (tips, procedures, activities).
- What criteria do you use to explain that this is a successful coordination activity?
- What method of coordination would be most effective in communicating a request to management? And vice versa, a request from management?

Summary

Although the interview was very perceptual, be vigilant as to whether the elements mentioned are generalized or not. Typical care process followed by the patient (normal case =% frequency) (problematic case =% frequency) or number of interveners on how much if the coordination mode changes according to the way of working of each. The delay caused by the modes of coordination is also important, since it directly affects the average length of stay of the patient. Impact other than in time. The perception of the impact of a variant on the normal coordination process can provide interesting information in terms of hospital culture, beliefs and the motivations of the person, as well as the quality of care provided. These sample questions are a guide, but the questions will be asked according to the topics brought by the person met. By presenting this guide and the research subject at the start of the interview, the person will understand the type of information sought. It will suffice to re-plan the grid to redirect the subject to the information sought. It is not necessary to pass the columns one by one, they are not technical and mechanical interviews. The grid is only a guide to remind the subjects which can be approached at different times.

Impact of the Orthopedist Involvement in the Management of Clinical Activities at Centre Hospitalier Universitaire (University Hospital Center, CHU) in Quebec, Canada

CONFIDENTIEL

Personal Information

Date : ____________________ Location: ____________________

Employee name: ____

Function: ____________________ Service : ____________________

Supervisor: ____

Hospital		Surgery		Care episode	
CHUL	☐	Total knee replacement	☐	Pre-admission	☐
Enfant-Jésus	☐	Total hip replacement	☐	Preoperative	☐
St-François d'Assise	☐	Hip fracture	☐	Perioperative	☐
L'Hôtel-Dieu de Québec	☐			Postoperative	☐

Diversification of clinical activities	Total replacement knee	Total hip replacement	Hip fracture
%			

Anecdotes, positive or negative, related to modes of coordination.

____

____

Coordination experiences (techniques and interaction)

Speakers	Administrative structures	Coordination mechanism	Rules and protocols	Informal agreement	Interactional dynamics

Perception of roles and work structures

Speakers	Preadmission	Preoperative	Peri operative	Operation	Postoperative

Involvement of physicians in the collective coordination process.

____

Opinion on variations in MDS between hospitals.

____
