# Supplementary material for: Orthopedist involvement in the management of clinical activities: a case study
Source: BMC Health Serv Res. 2021 Apr 1;21:299. doi: 10.1186/s12913-021-06299-2 (PMC8017788; doi:10.1186/s12913-021-06299-2)
Supplement: Supplementary file 2 — Additional file 2: Table S2. Levels of Orthopedic Surgeons’ Involvement in Clinical Activities, by Episode of Care and Other Clinical Staff Members’ Responses. [file 12913_2021_6299_MOESM2_ESM.docx]

**Table S2**: Levels of Orthopedic Surgeons’ Involvement in Clinical Activities, by Episode of Care and Other Clinical Staff Members’ Responses

| Clinical Activities,  by Episode | Status of Orthopedists’ Involvement and Challenges | Site | Other Clinical Staff Members’ Responses | Patient Outcomes  (Results of quality care for patients) |
| --- | --- | --- | --- | --- |
| **Daily surgery scheduling**   - Submission of surgery requests - Follow-up on surgery requests - Participation in surgery scheduling - Management of problematic cases | **Inactive**   - Feel no need to be involved because TKR, THR, and FH are programmable operations that do not call for much follow-up, except in cases of abnormality or emergency. - Have limited knowledge of patients’ medical records. - Pay little attention to scheduling procedures. - Prefer to use their own personal notebook rather than the hospital computer system programming for surgeries. - Do not follow the protocols, due to past culture of non-involvement in the surgery scheduling process. | A/C | - A head nursing (AHN) prepares the list of cases for the operating schedule, which is validated by the head orthopedist. - Duplicate work and additional examinations due to the high number of cases scheduled at the last minute and to the difficulty of reaching physicians. - AHN anticipate patient needs to shorten their stay and arrange meetings with other clinical staff members (physiotherapist, nutritionists, etc.). - Nurses call the patients on the list to inform them of the schedule and of the ambulatory unit, 2−3 hours before; nurses coordinate with the head of the operating room. - Administrators have greater control over daily surgery planning because institutional rules allow them to select and prioritize up to 50% of cases without consulting orthopedists. - Receptionists plan and arrange patient preparation, given their experience. - Various gaps in coordination put considerable pressure on surgery activities and lead to delay or even postponement of surgery. | - Surgeons’ lack of involvement has a number of consequences, including: (1) limited knowledge of patients, since they did not check the case list before the surgery date; 2) a high number of last-minute requests, which caused many delays, postponements, or cancellations, thus complicating surgery procedures and impacting the work of other clinical staff. For instance, the AHN had to change the order of priority, call other patients, or cancel the surgery, sometimes resulting in patients having to come back to the pre-operative clinic at a later date and even pass the necessary tests and consultations again, if the timeframe is exceeded. - Limited collaboration with other staff members increases stress around organizing and planning surgeries. - An increase in the number of patients transferred to other sites may further complicate patient care pathways. |
|  | **Contributory**   - Physicians decide whether to operate or not; then, the request is sent to the admissions department where a surgery schedule is set accordingly. - Orthopedists delegate management of the surgery schedule to the central planning unit, according to protocols, which they themselves co-developed. - Orthopedic surgeons prioritize their surgery requests on a scale of P1 (most urgent) to P4 (least urgent). | D | - The centralized surgery planning unit facilitates the selection of prepped cases (in the case of interchanged patients or cancellations). - Constant communication between the AHN in charge of the surgery schedule and the orthopedic surgeons may prevent low health performance. - When unforeseen situations arise, the AHN in charge of the surgery schedule changes the order of priority on the assumption that this change will be approved by the orthopedic surgeon. - Orthopedists delegate the task of scheduling dates for surgery, and instead set surgical priorities. - They also indicate the medical consultations required during patient preparation, have a fairly accurate idea of the cases on the list, and maintain easy communication with other health care providers. | - Orthopedic surgeons’ involvement in planning activities facilitate surgeries and had a number of advantages, including fewer postponements. - The surgery schedule is developed to consider the constraints of instrument sterilization. - A limited number of surgeries per day make for better control and eliminated overload by additional cases; it works very well with surgeons. - Despite a very full schedule (many emergencies on top of electives), an effort is made by medical team to cancel as few surgeries as possible; except in life-threatening situations, elective surgeries must occur before 5 p.m. while emergency surgeries are ongoing. |
|  | **Active**   - Orthopedists set the surgery date according to the severity of the case and ensure the planning process (availability of beds, etc.). - Surgery planning is entirely in their hands and they work with others for scheduling   orthopedists control the surgery case list and know patients’ medical records and the scheduled surgery date well.   - Except for oncology orthopedists, for whom patients are even more of a priority. - Orthopedists are also sensitive to the need to review and improve business processes. | B | - An interdisciplinary approach to the coordination process has greatly smoothed interpersonal dynamics. - Clinical staff members take all the necessary dispositions to better plan patients’ health pathways. - They sync with the OR coordinator before the patient arrives - They inform patients and the ambulatory care unit of the surgery schedule, 2−3 hours prior; in the case of cancellations, the surgeon will meet patients in the ambulatory care unit to give them another date. - Sometimes the schedule is drafted simultaneously: If there is cancellation, another patient can be selected from the patient bank. - Communication is much more oral with social workers, physiotherapists, and occupational therapists than written, as the objective is to not increase the number of unnecessary consultation requests. | - Physicians control the surgery case list and know patients’ medical records and the scheduled surgery date well. - This involvement has a huge impact, by facilitating all team members’ tasks and improving overall health performance outcomes: (1) shortening patient stays; (2) speeding up patient recovering; (3) increasing patient satisfaction and health; (4) facilitating smooth planning; (5) promoting the use of new technologies to facilitate communication; and (6) encouraging innovation through further training and reading. - Coordination occurs at a weekly meeting of all stakeholders except doctors. |
| **Instrument management**   - Equipment orders and delivery - Transmission of information | **Inactive**   - The impact of orthopedic surgeons’ lack of involvement in instrument management was mitigated by other clinical staff members’ interventions. - Orthopedic surgeons only glance at instrument management orders and do not follow up on them. | A | - The AHN is in charge of overseeing instrument preparation to prevent errors in their ordering. - The RN-surgery documented each orthopedic surgeon’s operating procedures and created a form with insets containing the specifications of the various prostheses in use. - The instrument selection, sterilization, and arrangement process was hard work for nurses, since each orthopedist has their own preferences for prostheses. - RN-surgery’s knowledge of operating procedures supported the work of orthopedic surgeons, nurses, and AHN for material resources. - RN-surgery also identified problems, suggested appropriate correctives, and ensured the training of OR nurses. | - Their lack of involvement in instrument management causes orthopedists to request a high number of last-minute instrument changes. - High patient stress and bad experiences may be caused by surgery rescheduling, cancellation, or postponement prompted by the non-availability of the right instruments. - Non-formalized procedures undermine health performance outcomes. - Duplicated work and additional examinations are caused by the great number of last-minute surgery requests, possibly making instrument management harder to schedule. |
|  | **Contributory**   - Instrument management is incorporated into the centralized surgery planning protocol. - Orthopedic surgeons are vigilant so that everything complies with the protocol (of which they are co-developers). | D | - Clinical staff members’ participation in developing the surgery planning protocol was widely considered a great success, and this was attributed to a formalization of the instrument ordering process, which has clarified and designated instrument management tasks. | - The standardization of orthopedic practices in the site D greatly facilitates the application of the protocol and contributed to health performance outcomes. |
|  | **Active**   - Orthopedic surgeons order their prostheses themselves and follow up on these orders. | B/C | - A series of charts are drawn to depict the types of prostheses used by each orthopedic surgeon and describe their respective positions in the operating room. - Completed forms were drawn up for the sterilization and purchasing departments indicating each orthopedic surgeon’s requirements for each type of procedure. | - Orthopedic surgeons’ involvement helps formalize instrument management. - It also reduces the number of postponements and last-minute instrument changes. |
| **Patient preparation**  (i) Hip fractures (**HF**)   - Medical consultation requests   (ii) Elective cases  (**TKR** and **THR**)   - Patient evaluation - Complex case management - Transmission of information | **Inactive**   - Surgeons work independently. - Some orthopedic surgeons made sure that a routine consultation had indeed taken place, while others simply submitted the surgery request and assumed that the consultation had happened. | A/C | - The bed manager postponed difficult cases to a later surgery date, to give the various specialists time to do their intra-operational activities. - The situation was more complicated for patients of orthopedic surgeons on the mobile team. Since there was no agreement among physicians, management was turned over to the pharmacist on duty (for preparation of the medication profile) and to a general practitioner (for patient assessment and drug prescription). | - The situation becomes complicated when the patient is transferred from another emergency room, since prior preparation may not have been done. - However, orthopedic surgeons also intervene if necessary. - Medical consultants do not assign slots of time for meetings with patients; also, it is difficult to reach some orthopedic surgeons who do not have offices on site. |
|  | **Contributory**   - Hip fractures and elective surgeries are managed by emergency physicians on the trauma team; patients are managed by the team. - The orthopedic surgeons are part of medical group management structure that handled all clinical staffs’ income, redistributed sums as agreed, and governed the surgery department’s internal operations. | B/D | - When preparing elective cases (TKR and THR), patients’ medical and pharmacological assessments are carried out by an internist in sites B or by an anesthesiologist in site D. - Under agreements between medical consultants (internists, anesthesiologists and cardiologists) and orthopedic surgeons, specific slots of time are assigned for orthopedic patients. | - Proposed innovations are carefully examined, analyzed, accepted, or rejected by the group. - Medical group management structure handles all the medical staff’s cases, redistributes sums as agreed, and governs its internal operations. |
| **Intra-operative phase**   - Surgery time management - Work organization in the operating block - Clinical practices improvement - Daily management of clinical activities | **Reactive**   - Orthopedic surgeons do not get involved in committees responsible for the daily management of clinical activities. - They simply made note of the instructions and orders issued by these committees. | A/C | - The clinical staff was constantly trying to track down attending physicians to obtain necessary medical prescriptions. - Last-minute changes to the surgery schedule entail additional time for patient preparation. - To avoid pointless consultations, a multi-site interdisciplinary team developed a data collection form to be completed by the patient. - For patients transferred from Site A due to bed shortages, nursing staff had to check that no information was missing and often had to request that consultations in internal medicine be redone. | - To avoid pointless consultations, a multi-site interdisciplinary team developed a data collection form to be completed by the patient. - The data collection is important for issuing the prescriptions required for patient preparation and to identify patients whose return home will be difficult. |
|  | **Contributory**   - Orthopedic surgeons’ involvement in the daily management of clinical activities was contributory at sites B and D. - At Sites B and D. At Site D, the medical group management structure, combined with interpersonal dynamics conducive to communication between orthopedic surgeons and other clinical staff members, facilitated their involvement. | B/D | - Group practice (by internists, anesthesiologists, cardiologists, and orthopedists) of exchanging cases, thus allowing each surgeon to specialize in a particular type of procedure. - Orthopedic surgeons are not part of a medical management group structure. - Intraoperative activities are incorporated in the centralized surgery planning protocol at Site D. | - Orthopedic surgeons have contributory involvement, the procedure goes smoothly due to agreements concluded between orthopedic surgeons and internists for pre-operative patient assessment and with cardiologists and anesthesiologists for the assignment of fixed slots of time for consultations. - At these two sites, orthopedic surgeons also contribute to work management organization and management activities by participating in clinical quality improvement committees. |
| **Post-operative patient follow-up**   - Routine medical visit - Patient discharge - Intervention in the event of complications | **Inactive**   - Orthopedic surgeons’ lack of involvement at two sites undermines post-operative activities there. - At Site C, one orthopedic surgeon follows up with these patients himself. - At Site A, three out of four hip fracture patients are managed by internists. | A/C | - For hip fractures, the discharge decision is often made by interdisciplinary collaboration with geriatricians or internists. - Patient monitoring is carried out by the resident internist and orthopedist. - Orthopedists simply refer the case to internists. | - To offset the absence of orthopedic surgeons, the clinical staff has attempted to formalize and standardize care processes via clinical tools, including care pathways and preprinted individual prescriptions. |
|  | **Contributory/Active**   - Orthopedic surgeons have delegated this task to orthopedic residents. - Orthopedic surgeons regularly use preprinted individual medical prescriptions. | B/D | - For hip fractures, the discharge decision is often made by general practitioners in the trauma program and residents. - Health care providers also emphasized the importance of a standardized approach. By constantly repeating “the patient has to leave in four days” (D3), they tried to make each team member aware of the importance of reviewing practices to achieve this discharge objective. - Orthopedic surgeons refer cases to residents at the slightest complication, which causes delays in patient discharge. - A checklist has been incorporated as standard practice. | - At Site B, care pathways are not updated, which undermines health performance outcomes (long hospital stays). - At Site D, an interdisciplinary team updated the care pathway and created proactive patient discharge criteria form to promote follow-up to episodes of care. - The secret was to involve care providers in the change process and provide constant reinforcement. - Patient coordinators are also integrated into the patient care unit. - The culture of cooperation and teamwork has developed over the years. |
| - **Inactive:** Not involved in developing management methods and of response to directives and requests - **Reactive:** Available if needed and responds to requests - **Contributory:** Contributes to activities to develop management and intervention methods, and delegates application to other clinical staff members; however, orthopedic surgeons keep a close eye on planned activities and intervene if necessary - **Active:** Shows active participation in the development and application of management methods | | | | |
